# Neuroimaging, neuropsychological and psychopathological findings in Medication Overuse Headache (MOH) before and after detoxification

**DOI:** 10.1186/1129-2377-14-S1-P172

**Published:** 2013-02-21

**Authors:** P Pozo-Rosich, G Cuberas-Borros, S Gomez-Baeza, C Lorenzo-Bosquet, C Jacas, J Alvarez-Sabin, C Roncero, C Roncero, J Castell-Conesa

**Affiliations:** 1 Headache and Neurological Pain Research Group, Neurology Department, University Hospital Vall d’Hebron, Barcelona, Spain; 2 Nuclear Medicine Department, University Hospital Vall d’Hebron, Barcelona, Spain; 3 Outpatient drug clinic, Psychiatry Department, University Hospital Vall d’Hebron, Barcelona, Spain; 4 Nuclear Medicine Department, University Hospital Vall d’Hebron, Barcelona, UK; 5 Department of Psychiatry and Legal Medicine, Universidad Autonoma de Barcelona, UK; 6 Department of Psychiatry and Legal Medicine, Universidad Autonoma de Barcelona., Spain; 7 Nuclear Medicine Department, University Hospital Vall d’Hebron, Spain

## 

Our hypothesis is that in MOH there are metabolic anomalies in brain areas which are implicated in drug dependence and impulse control.

## Objective

To identify changes in the dopamine D2 receptor in the striatum and, evaluate neuropsychological and psychopathological traits in MOH (before and after detoxification), chronic migraine (CM) and episodic migraine (EM) patients. Method: We studied 18 MOH, 14 CM, 18 EM patients and 5 controls using Iodine-123-iodobenzamide (IBZM) brain SPECT. MOH patients were studied before and, one and six months after detoxification. Neuropsychological and psychopathological traits were evaluated using standard tests and auto-applied scales.

## Results

SPECT quantification using spatially normalized images to an IBZM template showed the following mean and standard deviations for striatal ratios: 1.77±0.15 (Controls), 1.73±0.25 (EM), 1.61±0.10 (MOH) and 1.53±0.10 (CM). MOH and CM showed a similar downregulation of D2 receptors different to the D2 ratios seen in EM and controls. Statistical differences were found between controls/EM and CM (p<0.05). No differences were found in the serial SPECT’s done to the MOH patients. Significant differences were found between EM and MOH regarding an anxiety disorder and in tests measuring attention, executive function and verbal memory. There were also differences between CM and MOH patients. MOH patients’ quality of life and neuropsychology traits clearly improved after 6 months, with a lower medication intake (67.31 to 8.21pills/month).

## Conclusion

There were two different groups regarding the IBZM-SPECT results MOH/CM vs. EM/controls. MOH was different than CM/EM neuropsychologically and psychopathologically. MOH patients clinically improve after detoxification even if their IBZM-SPECT does not. So, maybe CM and MOH patients are clinically different because of their cultural and personal pain-coping strategies; and lower levels than expected of medication intake or a longer history of headpain, could alter D2 receptors of the brain.
